# Examining the Spillover Economic Impacts of Caregiving Among Families of Children With Medical Complexity to Inform Inclusive Economic Models: Qualitative Study

**DOI:** 10.2196/60666

**Published:** 2024-12-30

**Authors:** Jessica Keim-Malpass, K Jane Muir, Lisa C Letzkus, Eleanore Scheer, Rupa S Valdez

**Affiliations:** 1 Division of Pediatric Hematology-Oncology Department of Pediatrics University of Virginia School of Medicine Charlottesville, VA United States; 2 School of Nursing University of Pennsylvania Philadelphia, PA United States; 3 Division of Developmental Medicine Department of Pediatrics University of Virginia School of Medicine Charlottesville, VA United States; 4 Department of Systems and Information Engineering University of Virginia School of Engineering Charlottesville, VA United States; 5 Department of Public Health Sciences University of Virginia School of Medicine Charlottesville, VA United States

**Keywords:** caregiving, children with medical complexity, social network, qualitative, self-management, care coordination, economic evaluation, spillover, economic model, care, mobile phone

## Abstract

**Background:**

Children with medical complexity represent a heterogeneous group of children with multiple chronic health care conditions. Caregivers of children with medical complexity experience a high intensity of caregiving that is often variable, extends across several networks of care, and often lasts for the entirety of the child’s life. The spillover, or indirect, economic impacts of caregiving are understudied in the context the family units of children with medical complexity. There have been recognized limitations to the sole use of quantitative methods when developing economic models of disease, because they lack direct caregiver voice and context of caregiving activities, and existing methods have been noted to be ableist.

**Objective:**

This study aimed to explore the economic spillover impacts of caregiving among families of children with medical complexity using their own words and perspectives, with the intent of expanding caregiver-centered perspectives when developing economic models.

**Methods:**

This study was a secondary analysis of a qualitative study that was conducted to examine family management practices among caregivers of children with medical complexity and their social networks. Caregivers of children with medical complexity were recruited through a pediatric complex care clinic at an academic medical center in the mid-Atlantic region, United States. This study used inductive qualitative descriptive methods and a template to define features of the person impacted and to define the economic construct as either a direct or indirect (spillover) cost.

**Results:**

A total of 20 caregivers were included in this study. Perspectives from the caregivers of children with medical complexity revealed several key themes: (1) time lost from employment, impacting the primary caregivers; (2) physical and mental health impacts, impacting the child themselves, siblings, and the primary caregivers; (3) impacts to leisure activities and self-care, impacting the child themselves, siblings, and the primary caregivers; and (4) impacts to the social network or social capital.

**Conclusions:**

The themes described can be operationalized into inclusive family-centered models that represent the impacts of caregiving in the context of the family units of children with medical complexity. The use of qualitative methods to expand our development of quantitative economic models can be adapted to other populations where caregivers are involved in care. Caregivers can and should have an active voice in preference-based assessments that are operationalized in economic contexts to make them more inclusive.

**International Registered Report Identifier (IRRID):**

RR2-10.2196/14810

## Introduction

Efforts to improve the value of health care services for patients, families, and payers have increasingly incorporated health economic evaluations that measure the costs and outcomes of health care services and interventions through causal methods, cost-effectiveness, cost-utility, or decision analyses [1,2]. Often underrepresented in these approaches are the inclusion of spillover effects, or the unintended economic externalities of studied disease states, interventions, or care models to relational entities beyond associated health outcomes or direct costs of health care services, such as the health impacts to family members and caregivers [3,4]. As such, the measurement of spillover effects captures a more comprehensive depiction of the cost outcomes and health impacts of affected groups and the potential impact on society [5].

Spillover effects are unintended, can be positive or negative, and can impact an entity beyond the initial person of interest. Studies evaluating spillover effects have advanced evidence on, for example, the spillovers of nurse burnout to emergency department patient outcomes, mental health burdens for caregivers of children with autism, the health impacts of caregiving, and parents’ health insurance status on a child's school absenteeism [6-9]. Despite multifaceted approaches to evaluating spillover effects, their inclusion in health economic models is sparse and largely absent in literature involving children [10,11].

Economic models using a cost-effectiveness framework are often used in medical decision-making contexts to represent both present and future costs and benefits in evaluating novel therapeutics or health technologies [12]. A defining outcome of most cost-effective studies is the concept of the quality-adjusted life-year (QALY), which is the evaluation of perfectly healthy life-years after accounting for adverse effects of the condition and associated treatment. QALYs have been widely criticized for not equally valuing intervention benefits for those patients who are disabled compared with a nondisabled population [13]. This inherent perspective in quantitative valuation methods favoring nondisabled people likely contributes to societal structural ableism [14]. QALYs also only capture a small subset of potential benefits and ignore other more holistic elements of value, such as the value of equity, the value of hope, reduction in uncertainty, scientific spillovers, and so on [12]. Within this framework, economic spillover impacts on caregivers are rarely considered as elements of benefits and costs, and impacts on other members of the family and social network unit, like siblings, grandparents, and close friends, have not been widely reported in the economic literature. This lack of inclusion in economic decision-making models reduces the ability to make decisions that include the family context.

Children with medical complexity represent a heterogeneous group of children with multiple, chronic health care conditions that frequently use the health care system [15,16]. Children with medical complexity often require technology at home through the use of home-based mechanical ventilation, feeding interventions through gastrostomy tubes, and home-based intravenous infusions [17,18]. Because of the intensity of care, the unpredictable nature of their disease course, and complex multimorbidity, much of the actual care of children with medical complexity occurs in the home and community-based settings [19]. Furthermore, many of the interventions of care require ongoing family management and communication across members of the caregiving network (family, friends, home-based nursing staff members, and members of the child’s specialist providers and health care teams) [20].

There is substantial evidence documenting the economic impacts of caregiving, with most of the evidence focused on caregivers of older adults [4,9,21] and fewer focused on caregivers of children with medical complexity [11,22,23]. Economic effects among caregivers have been predominantly conceptualized as the direct valuing of caregiver time and, thus, underestimate opportunity costs, impacts on physical and mental health, impacts on employment, and impacts on other members of the household [21]. In general, studies demonstrate that unpaid caregivers are less likely to be used, are more likely to cut back on education, take more unpaid time off of work, work fewer hours, and are more likely to quit a job [21].

Surprisingly, caregiving costs are frequently not included in pediatric economic models despite the central nature of direct caregiving activities to children’s health [10,11]. A 2023 systematic review assessing the inclusion of family spillover impacts in pediatric cost-utility analysis found that out of 878 pediatric cost-utility analyses, only 35 included any family spillover effects within the model development [11]. Including family or caregiver spillover impacts is critical in properly assessing the impact of a novel health intervention on the entire family unit, or when assessing the overall burden of disease. Further complicating this lack of economic evaluation of caregiving as it relates to children with medical complexity is that caregiving activities are often negotiated among parents or guardians as primary caregivers, but also extend into diverse informal caregiving networks, including extended family members and friends in the community [20,24]. Because of the broad expanse of caregiving activity, broadly classified as family management of children with medical complexity, the spillover economic impacts are likely more diffuse and should include impacts to other children or siblings living in the house. Second, caregivers may perform caregiving activities throughout the entire life of children with medical complexities, as the child may still require significant caregiving support into adulthood.

There have been recognized limitations to the sole use of quantitative methods for economic evaluations, and there is emerging epistemological diversity in support of qualitative methods that can underpin a holistic approach to family- and caregiver-centered perspectives [3,25-28]. Expanding traditionally quantitative economic modeling frameworks to explicate family and caregiver benefits and costs of caregiving are necessary to make equitable individual, system, and societal decisions. By engaging with communities directly and understanding the experiences of caregiving through their own words and perspectives, we can include the spillover impacts they describe as important within economic models. Herein, we conducted a secondary analysis of a qualitative study assessing the negotiation of self-management tasks among caregivers of children with medical complexity and their social networks, to develop a broader understanding of the economic spillover impacts of caregiving for children with medical complexity using their own words and perspectives [20,24].

## Methods

### Design and Participants

Details of the primary qualitative study design and involvement of members of the social network can be found elsewhere [20,24,29]. Caregivers were recruited in a mid-Atlantic pediatric complex care clinic through purposive sampling. Caregivers could be included if they were primary caregivers (either parent or legal guardian), were younger than 18 years of age, were English speakers, and lived in the same household as the children with medical complexity. Children with medical complexity classification was defined using the Center of Excellence on Quality of Care Measures for Children with Complex Needs, because the complex care clinic that we recruited from uses this definition for referral patterns. By this definition, we identified children with medical complexity as those younger than 21 years with chronic conditions impacting 2 or more body systems, requiring resources beyond what is typical for most children, and relying on ongoing care management [30].

Primary caregivers were the focus of this secondary analysis. Clinical and demographic characteristics of the participants were collected so that the investigators could sample to ensure diversity in the types of children with medical complexity (body systems impacted, age, and technology dependence). Clinical characteristics were self-described by the caregivers themselves. Health literacy was assessed through a validated 3-question screen [31]. The interview guide was developed based on a theoretically-driven perspective to highlight the self-management experiences across families of children with medical complexity within diverse social networks and contextual environments [24]. The interviews were conducted from October 2019 through March 2021, which required flexibility in study conduct due to the evolution of the COVID-19 pandemic. Interviews were conducted over the phone, either in one more extended session or multiple shorter sessions throughout the week, based on the preference of the primary caregiver. While the interviews were conducted in a cross-sectional manner, either representing a singular day or week in time as a caregiver, the team used approaches from the interview guide to elicit longitudinal perspectives, both historically from the child’s birth and thinking to the future care, on implications of the children with medical complexity. Interviews were transcribed verbatim and were approximately 40-75 minutes in length.

### Analysis

Analysis was guided by qualitative description and thematic analysis, in which inductive approaches were applied to the entire dataset of primary caregiver interviews [32,33]. The first phase of analysis involved reading the transcripts and jotting memos of relevant contextual information related to the interview. The initial coding strategy was open based on elements of direct and indirect economic consequences and applied to the entire dataset. An a priori analytic template that identified direct versus indirect economic impacts guided the analysis informed by economic spillover literature [5,34]. The next phase involved transforming open codes into categories that may encompass several codes. In the final stage, a thematic analysis used underlying economic constructs, which aligned a final theme with the descriptive elements of the template analysis. This process allowed for themes conceptualized as model inputs in future economic models, outlining the people impacted and direct versus indirect or spillover costs. Attention to rigor and trustworthiness were handled through documentation of key analytic decisions, reflexivity practices including reflection on assumptions and positionality across roles (eg, researcher, clinician, and family member), epistemological perspectives of economic models, and review of the final themes by all members of the study team [35].

### Ethical Considerations

The University of Virginia Institutional Review Board for Social and Behavioral Sciences approved this study (SBS 2182). Informed consent for each study participant was obtained verbally. Participants were given a US $40 gift card following the conclusion of the interviews to compensate for their time and energy. Privacy was maintained by deidentifying interviews and linking by participant ID code.

## Results

### Participant Characteristics

A total of 20 primary caregivers of children with medical complexity were included in this analysis. Across the sample of 20 caregivers of children with medical complexity, the participants were on average 34.9 (SD 7.9) years of age; they were mostly female (18/20, 90%), White (17/20, 85%), and with at least some college education or more (14/20, 70%; Table 1). The most common medical conditions that parents reported of their children (not mutually exclusive) were cerebral palsy (8/20, 40%), congenital anomaly or genetic syndrome (7/20, 35%), and behavioral or mental health (7/20, 35%). Half (10/20, 50%) of the sample had adequate health literacy, and the majority (16/20, 80%) of the sample had home and phone access to the internet.

**Figure 1:**
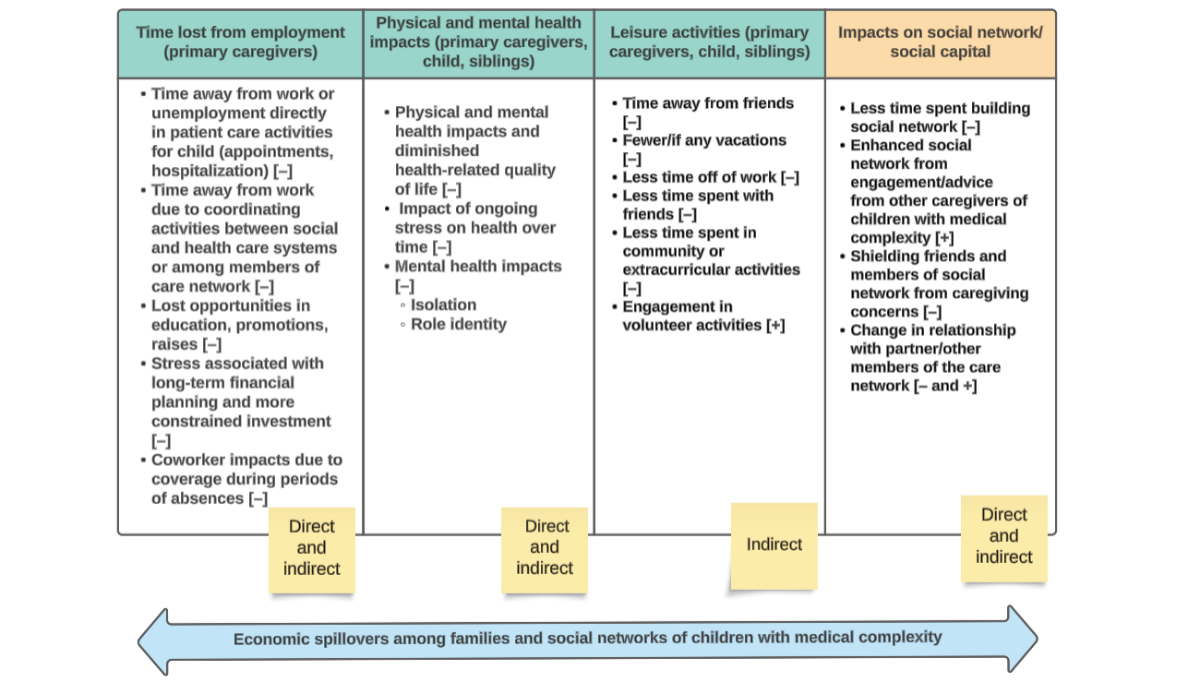
Representation of direct and indirect spillover economic impacts of children with medical complexity on caregiver and family units.

The thematic analysis of the participant interviews resulted following themes: (1) time lost from employment, impacting the primary caregivers; (2) physical and mental health impacts, impacting the child themselves, siblings, and the primary caregivers; (3) impacts to leisure activities and self-care, impacting the child themselves, and the primary caregivers; (4) impacts to the social network or social capital. The themes described can be operationalized into inclusive family-centered models that represent the impacts of caregiving in the context of the family units of children with medical complexity (Figure 1). We also developed a hypothetical case exemplar to demonstrate how spillover impacts of caregiving can be included in economic models.

**Table 1:** Participant and child characteristics.

Participant characteristics (N=20)		Values
**Age (years), mean (SD)**		34.9 (7.9)
	Missing, n (%)	2 (10)
**Gender identity, n (%)**		
	Female	18 (90)
	Male	2 (10)
**Own cell phone, n (%)**		
	Yes	18 (90)
	No	0 (0)
	Missing	2 (10)
**Own smartphone, n (%)**		
	Yes	17 (85)
	No	1 (5)
	Missing	2 (10)
**Highest level of education, n (%)**		
	Less than high school	1 (5)
	High school graduate or GED^a^	3 (15)
	Some college	9(45)
	2-year college degree	1 (5)
	4-year college degree	4 (20)
	Missing	2 (10)
**Race, n (%)**		
	White	17 (85)
	Black	2 (10)
	Missing	1 (5)
**Health literacy, n (%)**		
	Adequate	10 (50)
	Marginal or limited	8 (40)
	Missing	2 (10)
**Access internet, n (%)**		
	Home and phone	16 (80)
	Phone only	2 (10)
	Missing	2 (10)
**Medical conditions of the child (not mutually exclusive), n (%)**		
	Prematurity	5 (25)
	Chronic lung disease	5 (25)
	Cerebral palsy	8 (40)
	Epilepsy	6 (30)
	Brain tumor	1 (5)
	Congenital heart disease	3 (15)
	Feeding difficulty or poor weight gain	3 (15)
	Congenital anomaly or genetic syndrome	7 (35)
	Behavioral or mental health	7 (35)
	Endocrine	1 (5)
**Technology dependence of child (not mutually exclusive), n (%)**		
	Home oxygen requirement	3 (15)
	Gastrostomy tube	3 (15)

^a^GED: General education diploma.

### Theme 1: Time Lost From Employment

The first theme representing time investment in caregiving (Textbox 1 includes qualitative exemplars), encompasses several categories including time involved in the coordination of direct care, energy in financial navigation (reimbursement and insurance coverage), time required for information seeking, and the requirement for future financial planning (requiring both time and emotional energy). This theme also includes both direct and indirect or spillover economic implications. The direct financial impacts are elements such as time away from work due directly to patient care activities, coordination of care, financial and disease navigation, and so on. In addition, there may be interruptions to employment, loss of employment, opportunity costs from loss of promotion, raises, and so on. One caregiver describes as follows:

Well, I had a job before he came home, and then once he was home, I didn’t go back.

Participant 8

Another caregiver said the following:

I don’t work outside the house. My plan was to go back to work after she was born, but once we realized all of the doctor’s appointments and therapy appointments, it was just gonna be too difficult. I needed to be home to be her caregiver.

Participant 9

Participants describe the mental energy and countless hours spent in the context of information seeking and communication between medical teams and members of the social network. Numerous participants also discussed the time spent on future financial and long-term planning to ensure care coverage of their child for both the near and distant future. There are indirect economic implications to this theme as well. One example is the stress associated with short and long-term financial planning, which impacts mental and physical health and constrains investment opportunities. Finally, there are indirect impacts on the socio-organizational work environment surrounding caregivers, who are able to remain in the workforce but may experience interruptions within their workday or unexpected days off of work. Coworkers may have to cover more duties or feel resentful of the caregiver, which could negatively impact the work team.

**Qualitative exemplars: time investment (time away from employment for direct caretaking).**

**Theme**

- Time lost from employment

**Categories**

- Coordination of direct care
- Energy in navigation of reimbursement, insurance, and coverage
- Information seeking
- Future financial planning (time)

**Exemplars**

- *[We have to take turns with appointments because of work] Between therapies and doctor appointments, yup. There’s like ten every month [rotates with partner and mother].* [Participant 1]
- *They offer support when they’re visiting. They offer emotional support to my wife and I on phone calls or if we go to visit them, but big‑picture plan is just us.* [Participant 5]
- *I call them because no one knows. Yeah. There’s no making sense of it. There’ve been times where I have called between the office billing for a service and our primary insurance and then Medicaid 6 or eight times and gotten a different story every single time. Then suddenly magically, something resolved and no one knows why. I pretty much resigned myself to a couple full days on the phone and try not to cry.* [Participant 6]
- *The insurance especially when he [child with medical complexity] was first—when he first came out of the hospital, part of the reason why I didn't go back to work as soon as I—we had gotten to a point where we were ready for me to go back to work, but I took an extra—I took a lot of extra time because I was dealing with all of the insurance and everything else and trying to get him on Medicaid and all of the other things that went about with. Right now, a little bit of that has calmed down because we’re—he’s enrolled in Medicaid. A lot of that is set up now. In the beginning, it was figuring all that out, figuring out what he could be eligible for, fighting the insurance companies on things until we were on Medicaid and everything else that had to happen, made it so that I lost a lot of—I'm an hourly employee, so it made it so that I lost a lot of hours at work and days at work and income.*[Participant 7]
- *There were coworkers [that helped]. There’s the employers who gave me some flexibility. There’s the coworkers who stepped in to cover us in certain ways. There was the support of friends and family and community who came to the hospital to give us support, or family who came and stayed with us to help us immediately post‑op.* [Participant 7]
- *Hours and hours of phone calls and pressure and talking to people and different organizations. There was some help from care coordinators through early intervention and social services and some—yeah. We reached out and got a little bit of help from a number of people, but it’s—I don’t know if some of it was just because of the unique situations of his condition, but it also—we felt like we were very lucky that both of us were able to take time off work and were able to invest the time in making the phone calls, doing the research, getting him evaluated by social services, getting him evaluated by early intervention, all of these programs, which I don’t know that—we were able to do all of that because we were very motivated, and we were taking the initiative. I didn’t feel like the support system that’s out there was really very aggressive at finding ways to help us necessarily*. [Participant 7]
- *Well, I had a job before he came home, and then once he was home, I didn’t go back.* [Participant 8]
- *I don’t work outside the house. My plan was to go back to work after she was born, but once we realized all the doctor’s appointments and therapy appointments, that thing, it just was gonna be too difficult. I needed to be able to be home to be her caregiver.* [Participant 9]
- *We are constantly planning for the future in terms of financially, trying to prepare for the day that my husband and I no longer can take care of her.* [Participant 9]
- *Financially, yes, we are always preparing for the future. In terms of a plan while I’m still living, and I’m still capable of taking care of her, that’s the plan. Even in five short years, we have learned that this life of raising a child with medical complexity, the only consistent thing about it is its inconsistency. Things change constantly. It doesn’t really matter how we plan. A lotta times it typically doesn’t go to plan. That’s not necessarily to say that it changes in a bad way. Sometimes we get surprised, and we get blessed with something that we didn’t see coming, something as simple as a new waiver that we qualify for.* [Participant 9]

### Theme 2: Physical and Mental Health Impacts

The next theme broadly encompasses the physical and mental health impacts of chronic care and uncertainty on the health-related quality of life of children with medical complexity, siblings, and primary caregivers (Textbox 2 includes qualitative exemplars). The direct physical and mental health impacts can be represented in health economic models. One qualitative exemplar highlights this in the following way:

I have Crohn’s disease that whenever I get too stressed it starts to flare up. So it’s pretty much a circle. If she’s sick, I get sick because I get too stressed.

Participant 1

Feeding much of these experiences are the chronic and uncertain nature of the illness trajectory, the changes in role identity both within and outside of the family, and the mental health impacts of the isolation caused by chronic caregiving. As described by 1 participant:

It’s stressful. It’s difficult. It’s exhausting. It’s nonstop. There are days that are significantly better, there are time periods that are better, but those are still stressful and still tiring and everything.

Participant 7

There are several indirect or spillover impacts especially as it relates to the chronic nature of physical and mental health impacts such as the ability to participate in school, attend work, or engage in the community.

**Qualitative exemplars: physical and mental health impacts.**

**Theme**

- Physical and mental health impacts

**Categories**

- Chronic illness as caregivers
- Mental health: isolation
- Mental health: role identity
- Mental health: emotional energy future planning

**Exemplars**

- *[I have] Crohn’s disease that whenever I get too stressed it starts to flare up. So it’s pretty much a circle. If she’s sick, I get sick because I get too stressed.* [Participant 1]
- *I think, I mean, I have people who are in my social network who are helpful with the kids and that’s wonderful. I think having people that aren’t necessarily helping with the kids, that are just good with the kids, that enjoy them and enjoy being around, that’s a pretty huge thing. Because it’s very easy to feel isolated when you’re a caregiver*. [Participant 6]
- *Because that reminds me that I’m not just [child with medical complexity]’s mom and I’m not just the caregiver. There has to be balance*. [Participant 6]
- *It’s stressful, difficult, exhausting, and nonstop. There are days that are significantly better, and there are time periods that are better, but those are still stressful and still tiring and everything. They’re just less so than the bad days.* [Participant 7]
- *I don’t know. We just don’t know where [child with medical complexity]’s gonna be when he’s an adult. We have issues with him learning. One day he can do certain things, and it’s another day he doesn’t know any of what—he doesn’t retain what he’s learned. We have already made arrangements if something happened to myself and my husband that our older son—if something were to happen to us by the time they’re 21, he would take over that care. It’s just a what if.* [Participant 18]

### Theme 3: Impacts on Leisure Activities and Self-Care

Many participants described the challenges of relaxing with friends, taking time for themselves, and planning activities for leisure, such as vacations (Textbox 3 includes qualitative exemplars). The economic implications of this are predominantly in the indirect or spillover context, as the lack of time to be by themselves, engage in leisure activities, and lack of time or resources for self-care directly contribute to the physical and mental health impacts of care and can certainly contribute to overall well-being at work and school. One participant described as follows:

All her [child with medical complexity] medication was not working well because she needed an adjustment on her medication plus the machine that we take down there broke on me when we were down there so it was a lot of stuff together and it completely ruined our vacation…since then we never take a vacation again.

Participant 1

Uniformly, none of the participants described any leisure activities, hobbies, or self-care activities that they were able to participate in.

**Qualitative exemplars: impacts on leisure activity.**

**Theme**

- Impacts on leisure activities and self-care

**Categories**

- Primary caregivers, child, and sibling impacts

**Exemplars**

- *All her [child with medical complexity] medication wasn’t working well because she needed an adjustment on her medication plus the machine that we took down there broke on me when we were down there, so it was a lot of stuff together and it completely ruined our vacation so yeah. That was the first thing that I can think about. Since then, we never take a vacation again just because we don’t want none of that to happen again.* [Participant 1]
- *The other thing we notice is that if there’s—let’s go with a family picnic or something—other parents will kind of hang out around the table, having a drink, eating food, chatting with each other, things like that, while their kids run around and play and do all sorts of stuff. We miss out on that social interaction because we can’t just sit back and let him go. We have to be sometimes physically helping him, other times just watching him in a way that other parents don’t need to.* [Participant 7]

### Theme 4: Social Network and Social Capital

Participants described significant changes in their social networks from both a direct and indirect or spillover perspective. Often social networks have narrowed from the people who were in their lives before the birth of children with medical complexity. One participant describes the nature of changed relationships within their own household:

Specifically it has changed the dynamic of our relationship. I rely on him for a lot more than I used to. I was always a very independent person. The toll that raising [child’s name] put on me and me being her full-time caregiver changed me into not such an independent person and really needing to lean on him. That was a big change for both of us that we had to adapt to. Yeah, I’d say my relationship with my husband is the one that’s changed the most.

Participant 9

Many of the participants were very self-aware of the reliance on the network and did not want them to be overburdened. The same participant describes it as follows:

We try to be as cognizant as possible-with our friends and families-not to wear anybody down too much with our issues.

Participant 9

Of note, these experiences were not uniformly negative in relation to members of the social network that were engaged after the birth of their children with medical complexity. In fact, there were several positive examples of how social networks have expanded in unexpected ways through connection with other families of children with medical complexity, and how they view the health care team as a social network and social capital expansion (Textbox 4).

**Qualitative exemplars: impacts on social network or social capital.**

**Theme**

- Social network and social capital

**Exemplars [participant]**

- *[My social network] has narrowed. We have less contact with people who we used to have more contact with. We have less time to maintain relationships with people. Even when we get to see people within our social networks, our interactions with them are typically a little bit shallower because a certain portion of our attention is always devoted to [child with medical complexity].* [Participant 7]
- *[My social network] has gotten smaller*. [Participant 8]
- *We try to be as cognizant as possible about—with our friends and families, not to wear anybody down too much with our issues.* [Participant 9]
- *[Positive impact] I’ve been really fortunate. I know a lot of people, theirs [social network] have changed significantly. Mine really haven’t other than growing. I’ve added people to them just through meeting people who have kids with special needs. Obviously, we have a lot more doctors and therapists and teachers and stuff or through us seeking out new activities for her [child with medical complexity] to participate in. We meet other people through those kinds of things. It’s grown. I’ve been fortunate that it hasn’t shrunk.* [Participant 9]
- *Yeah, I’d say my relationship with my husband is the one that’s changed the most.* [Participant 9]

### Hypothetical Case Exemplar of Using the Findings to Inform an Economic Model

A new randomized controlled trial (RCT) was recently published that demonstrated the efficacy of a family-centered, home-based dual nursing and social work model for children with medical complexity during the transition from the hospital to the home. The family-centered, home-based dual nursing and social work model included physical and mental health assessments for the children with medical complexity, caregivers, siblings, and caregivers’ older parents in the house, along with navigation services if referrals to additional medical providers were needed. The embedded social worker aided with completing the appropriate documentation for short- and long-term disability from caregivers’ places of employment. The social worker also provided resources for financial or insurance navigation services. Finally, they both provided connections for monthly in-person and virtual support groups for caregivers of children with medical complexity and another support group specifically for siblings of children with medical complexity. The RCT assessed the outcomes of the children with medical complexity, the caregivers, and the siblings. The study demonstrated that the children with medical complexity in the intervention arm had greater adherence to medication and therapies and lower rates of 30-day unplanned readmission. The caregivers in the intervention arm demonstrated improvement in mental health outcomes, had fewer days missed from work, had a lower proportion of caregivers having to leave work over 2 years, and spent less time on the phone with insurance companies. They also reported improvements in social cohesion and social capital because of the connection to other children with medical complexity families. Siblings in the household in the intervention arm demonstrated improvement in mental health outcomes and fewer days missed of school. The older parents in the household also reported improved physical and mental health outcomes.

The dual, embedded, home-based, nurse and social work model was costly given the amount of time in the home and the long travel for home-based visits. An economist wishes to demonstrate the cost-effectiveness of this intervention to assist health systems and payers in determining the economic value of this intervention.

**Figure 2:**
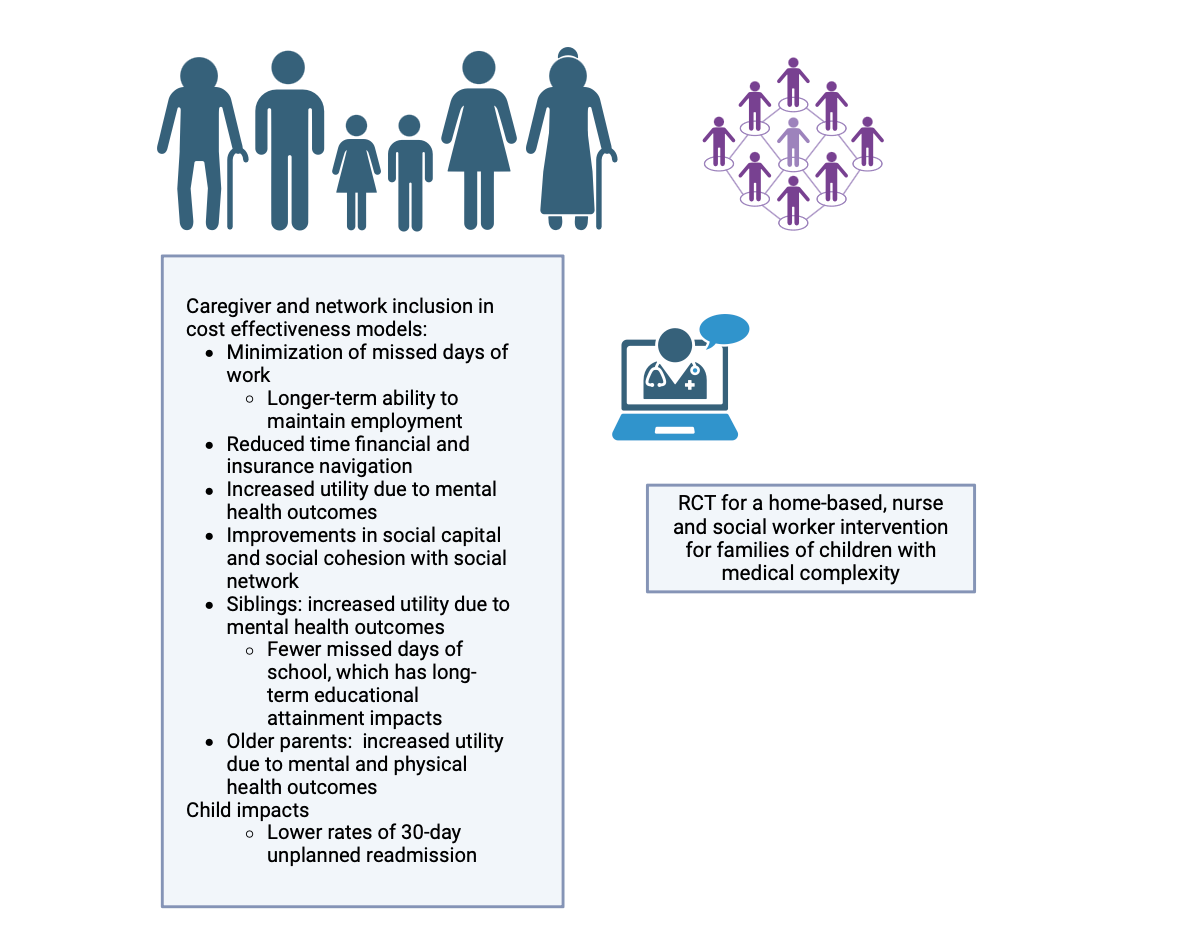
Broad view of economic impacts that can be included in a cost-effectiveness analysis based on the case exemplar. RCT: randomized controlled trial.

Figure 2 highlights the ways in which the outcomes of the RCT can be included in a cost-effectiveness analysis, which includes the children with medical complexity, caregivers, as well as older parents and siblings in the household (Figure 2).

## Discussion

### Principal Findings

This novel approach uses qualitative data elicited from direct caregivers and allows health economists and health services researchers to expand their modeling perspectives to include broader caregiver, sibling, child, and social network spillover impacts. This study provides guidance for exploring caregiver-centered perspectives on elements of caregiving that impact their health-related quality of life, ability to engage in work or education activities with implications for community cohesion, and changes to social networks. While direct economic caregiver impacts of families with children with medical complexity have been explored [36-39], there have been very few economic models eliciting the overall burden of disease in the form of indirect or spillover impacts. There has been much less attention paid to how to translate these impacts into quantitative economic models, such as cost-effectiveness models, that include model parameters beyond the child themselves or direct caregiving impacts.

Caregiving time constraints directly impact numerous aspects of life, including time away from employment, loss or reduction of employment, interruptions to education, and opportunity costs in relation to loss of career advancement or other related opportunities [5,21,23,40]. Physical and mental health impacts can be quantified through the assessment of disutility through QALYs, potentially leading to an ableist assessment that can be discriminatory [13,41,42]. Methods have been developed to offset the ableist lens of the QALY such as a measure of the health years in total [43] and the equal-value life-year [44]. Despite the expanded methodological approaches to offset ableist epistemological underpinnings, only direct costs and benefits related to the patients themselves tend to be included in these modeling approaches. Spillover impacts, as reported here, are rarely fully considered nor included in economic modeling efforts [3].

Our thematic findings coincide with conceptual definitions of family spillover effects that have been previously published [11]. A 2023 systematic review found that conceptual definitions of family spillover impacts include the health and nonhealth effects experienced by family members due to a child’s illness, disability, and treatment, encompassing physical and psychological health, emotional well-being, and quality of life [11]. They acknowledge that these impacts can not only be a result of direct caregiving tasks but also the result bearing witness to the enduring, decline, or death of their child [11]. Our findings extend previous definitions by also including social network members who engage in direct caregiving within these frameworks. Within this paradigm, family spillover impacts can be conceptualized as community spillover impacts.

Qualitative methods can extend traditional preference-based utility assessment to incorporate the lived experiences of those who are actively represented by these groups and expand our understanding of trade-offs of caregiving through the embedded context. They also can expand epistemological diversity by centering the perspectives of families caring for a child with medical complexity. As noted with the findings associated with the benefits of caregiving, caregivers of children with medical complexity often experience an expansion of a network of families in similar health situations and an expansion of a health care team that they generally trust to assist them with family management of their children with medical complexity. Positive aspects of caregiving are rarely considered with traditional economic perspectives, and there is immense opportunity to further assess the impact of network dynamics on economic outcomes and the effectiveness of health interventions. Even though our study did not demonstrate positive associations of caregiving on physical or mental health, there are certainly reported positive impacts of caregiving on stress and well-being [45]. This is one such example where reductionist utility assessments do not capture the full value of caregiving.

This work supports future methodological expansions of economic assessments to include a mixed methods perspective. Future research should work with caregivers to better understand their perception of the value of caregiving and then embed those perspectives into quantitative stated or revealed preference methods (such as discrete choice experience) to elicit utility values. Finally, qualitative methods can be used at the end of an economic modeling study again to present findings to caregiver stakeholders and get feedback on the quantitative model.

There are limitations to this analysis that we wish to acknowledge. While we used purposive sampling criteria based on child condition, the sample was still homogeneous with mostly White, female caregivers, with the majority having at least some access to at least some college education. The participants had levels of limited or marginal health literacy, which mimicked other real-world samples [46]. In addition, our study sample only included English speakers, which does not represent all families of children with medical complexity [47-49]. This study also was conducted in a single geographic region where state-based policies for children with medical complexity and home- and community-based care could impact the caregiving experience in various ways in terms of Medicaid coverage, access to durable medical equipment through Medicaid, and 1915/1115 home- and community-based waiver coverage. In addition, the sample was recruited from a comprehensive complex care clinic at an academic medical center, which connotes a high level of care coordination that likely takes place within the health system for children with medical complexity and their families. While we attempted to use approaches to garner longitudinal perspectives, interviews were still cross-sectional, taking place in either a single day or the course of a week. COVID-19 occurred in the middle of sample recruitment. Fortunately, the pandemic did not alter our study procedures, but it is unclear what impact the pandemic had on our recruitment or families’ abilities to participate or the narratives shared with us. Finally, we relied on several demographic characteristics from the interviews themselves, several items were not reported on in a way that allowed for harmonized reporting.

### Conclusion

Our work demonstrates that qualitative methods can provide an expanded perspective of traditionally quantitative economic models that relate to either assessing the effectiveness of health interventions directed toward children with medical complexity or the overall burden of disease for children with medical complexity and their families. Qualitative findings can be operationalized to help modelers build more inclusive and less ableist modeling perspectives. This method can and should be replicated in other populations where caregivers and social networks are involved in care. Caregivers can and should have an active voice in preference-based assessments that are operationalized in economic contexts. There is a defined need for interventions focused on caregivers of children with medical complexity focused on their own physical and mental health impacts, along with mitigation of financial toxicity. As demonstrated within this work, family spillover impacts can be conceptualized as community spillover impacts, given that members of the social network are also impacted. Finally, there is a critical need for this work to interface with those who are a part of the disability community, emphasizing the importance of understanding the intersection of other identities with disability in this context.

## Abbreviations

QALY: quality-adjusted life-year
RCT: randomized controlled trial
